# Report of Mortality in Buffalo for the Month of Dec., 1856

**Published:** 1857-02

**Authors:** C. L. Dayton

**Affiliations:** Health Physician


ART. VI.—*Report of Mortality in Buffalo for the month of Dec.*, 1856. By C. L. DAYTON, M. D., Health Physician.

| DISEASES. | No. | Males. | Females. |
|---|---|---|---|
| Accidens, | 1 | 1 | |
| Apoplexia, | 1 | | 1 |
| Burned, | 2 | 2 | |
| Bronchitis, | 2 | 2 | |
| Cancer, | 1 | | 1 |
| Cholera Infantum, | 1 | | |
| Cynanche Trachealis, | 6 | 2 | 4 |
| Cephalitis, | 3 | 3 | |
| Dentitio, | 2 | 1 | 1 |
| Diarrhœa, | 2 | 1 | 1 |
| Dysinteria, | 1 | | 1 |
| Debilitas Senilis, | 6 | 3 | 3 |
| Dissipatio et Delirium Tremens, | 3 | 3 | |
| Eclampsia, | 9 | 3 | 4 |
| Enteritis, | 1 | | 1 |
| Febris Typhoid, | 1 | | 1 |
| " Scarlatina, | 26 | 16 | 10 |
| " " Sequæla to, | 3 | 2 | 1 |
| " Ignotus, | 2 | 1 | 1 |
| Gastritis, | 1 | | 1 |
| Hydrops, | 4 | 2 | 2 |
| Hydrothorax, | 1 | | 1 |
| Hydrocephalus, | 4 | 3 | 1 |
| Hæmorrhagia Uterina, | 1 | | 1 |
| Hyperæmia Pulmonalis, | 1 | | 1 |
| " Cerebralis, | 1 | | 1 |
| Ignotus, | 16 | 6 | 6 |
| Icterus, | 1 | | |
| Morbus Brightii, | 1 | 1 | |
| " Cordis, | 1 | 1 | |
| Metritis, | 1 | | 1 |
| Marasmus, | 4 | | 1 |
| Narcosis, | 1 | 1 | |
| Paralysis, | 1 | 1 | |

REGISTER OF MORTALITY—Continued.

| DISEASES. | No. | Males. | Females. |
|---|---|---|---|
| Pertussis, | 1 | 1 | |
| Peritonitis, | 7 | 2 | 4 |
| " Lobular, | 1 | | 1 |
| " Puerperarum, | 1 | | 1 |
| Rubeola, | 1 | | 1 |
| Scrophula, | 1 | 1 | |
| Syphilis, | 1 | 1 | |
| " Congenital, | 1 | 1 | |
| Still-born, | 9 | | |
| Spina Bifida, | 1 | 1 | |
| Tuberculosis Pulmonalis, | 13 | 5 | 8 |
| Total, | 149 | | |

SEXES.

| | |
|---|---|
| Males, | 65 |
| Females, | 61 |
| Sex not given, | 23 |
| Total, | 149 |

AGES.

| | | | |
|---|---|---|---|
| Still-born, | 9 | Between 10 years and 20 years, | 8 |
| 1 day, | 3 | " 20 " " 30 " | 13 |
| 1 day and 30 days, | 7 | " 30 " " 40 " | 6 |
| Between 1 month and 6 months, | 15 | " 40 " " 50 " | 12 |
| " 6 months and 12 " | 9 | " 50 " " 60 " | 5 |
| " 1 year " 3 years, | 36 | " 60 " " 70 " | 5 |
| " 3 " " 5 " | 7 | " 70 " " 80 " | 5 |
| " 5 " " 10 " | 5 | " 80 " " 90 " | 1 |
| | | " 90 " " 100 " | 1 |
| Total number under 10 years, | 91 | Total number over 10 years, | 56 |
| Ages not given, | 2 | Total, | 149 |

NATIVITIES.

| | | | |
|---|---|---|---|
| American, | 68 | Prussian, | 1 |
| German, | 31 | French, | 2 |
| Irish, | 16 | Scotch, | 3 |
| English, | 3 | Holland, | 3 |
| Canadian, | 1 | Country not given, | 21 |
| Total, | | | 149 |

